# Effects of Adding Interferential Therapy Electro-Massage to Usual Care after Surgery in Subacromial Pain Syndrome: A Randomized Clinical Trial

**DOI:** 10.3390/jcm8020175

**Published:** 2019-02-02

**Authors:** Manuel Albornoz-Cabello, Jose Antonio Sanchez-Santos, Rocio Melero-Suarez, Alberto Marcos Heredia-Rizo, Luis Espejo-Antunez

**Affiliations:** 1 Department of Physiotherapy, Faculty of Nursing, Physiotherapy and Podiatry, University of Seville, 41009 Seville, Spain; malbornoz@us.es; 2 High Resolution Hospital, Andalusian Health Service, Utrera, 41710 Sevilla, Spain; jossansan@alum.us.es; 3 Department of Podiatry, Faculty of Nursing, Physiotherapy and Podiatry, University of Seville, 41009 Seville, Spain; rms-1000@hotmail.com; 4 Department of Medical-Surgical Therapeutics, Faculty of Medicine, University of Extremadura, 06006 Badajoz, Spain; luisea@unex.es

**Keywords:** electric stimulation therapy, manual therapies, musculoskeletal pain, pain assessment, range of motion, shoulder pain

## Abstract

Subacromial pain syndrome (SAPS) is a prevalent condition that results in loss of function. Surgery is indicated when pain and functional limitations persist after conservative measures, with scarce evidence about the most-appropriate post-operative approach. Interferential therapy (IFT), as a supplement to other interventions, has shown to relieve musculoskeletal pain. The study aim was to investigate the effects of adding IFT electro-massage to usual care after surgery in adults with SAPS. A randomized, single-blinded, controlled trial was carried out. Fifty-six adults with SAPS, who underwent acromioplasty in the previous 12 weeks, were equally distributed into an IFT electro-massage group or a control group. All participants underwent a two-week intervention (three times per week). The control group received usual care (thermotherapy, therapeutic exercise, manual therapy, and ultrasound). For participants in the IFT electro-massage group, a 15-min IFT electro-massage was added to usual care in every session. Shoulder pain intensity was assessed with a 100-mm visual analogue scale. Secondary measures included upper limb functionality (Constant-Murley score), and pain-free passive range of movement. A blinded evaluator collected outcomes at baseline and after the last treatment session. The ANOVA revealed a significant group effect, for those who received IFT electro-massage, for improvements in pain intensity, upper limb function, and shoulder flexion, abduction, internal and external rotation (all, *p* < 0.01). There were no between-group differences for shoulder extension (*p* = 0.531) and adduction (*p* = 0.340). Adding IFT electro-massage to usual care, including manual therapy and exercises, revealed greater positive effects on pain, upper limb function, and mobility in adults with SAPS after acromioplasty.

## 1. Introduction

Shoulder complaints are a common musculoskeletal disorder. The one-year prevalence of shoulder pain in the general population ranges from 5% to 47%, while the lifetime prevalence has been estimated up to 67% [1]. Work-related physical and psychosocial factors may be associated with onset and/or worsening of shoulder pain within the working-age population [2]. Among shoulder complaints, subacromial pain syndrome (SAPS) is the most common disorder that result in loss of function, increased pain sensitivity [3], and impaired quality of life, accounting for up to 70% of consultations in primary care [4]. SAPS is characterized by persistent pain around the acromion, which usually worsens during or after lifting the upper extremity [5], and embraces clinical diagnosis such as subacromial impingement and rotator cuff tears [6]. The clinical course of SAPS remains unclear, and previous evidence suggests that 50% of adults with chronic SAPS may only recover after 10 to 18 months of initial onset [7]. This leads to a considerable economic burden [8], due to absenteeism from work, productivity loss, and high expenditure for health care services [9].

A great diversity of conservative interventions, combining pharmacological and physical therapy treatments, is often used to decrease pain and enhance function in SAPS [5]. There exists; however, very limited evidence about the effectiveness of existing treatments to improve the functional limitations associated with this condition [10]. Amongst them, exercise therapy has been suggested as the core conservative treatment [11]. Likewise, the use of deep dry needling has shown to relieve shoulder pain [12], and ultrasound-guided injection therapy is widely used before surgery, with good outcomes in the short-term [13,14]. Surgical intervention is mainly indicated when pain and functional limitations persist after conservative measures, and for patients with clearly distinguished clinical signs [11,15]. Indeed, there is conflicting evidence about the efficacy of surgery compared to conservative approaches [16,17], or no treatment [15]. Despite that, the frequency of acromioplasty has dramatically increased in the last decades [18,19]. There is; however, scarce evidence about the most-appropriate post-operative intervention for SAPS, with exercise therapy showing good results [20,21]. 

Interferential therapy (IFT) is a highly popular treatment modality in the clinical setting, which involves crossing two medium frequency currents to generate a low-frequency beating effect in the deep tissues [22], and can be used alone or combined with massage [23]. Although IFT is purported to provide pain relief and increased blood flow to the tissues [24], there is still inadequate evidence to support its use as a sole intervention for pain management in musculoskeletal disorders in general [22], and in shoulder pain in particular [25,26]. Nevertheless, IFT as a supplement to other interventions has demonstrated advantages over placebo and control treatments for reducing musculoskeletal pain [27], although there are conflicting findings on this issue [28]. Current research also highlights the need for high quality clinical trials assessing the effectiveness of multimodal approaches for SAPS [29].

The study aim was to investigate the effects of adding IFT electro-massage to a two-week usual care protocol, compared with the usual care protocol alone, on pain intensity, upper limb functionality, and shoulder passive range of motion in adults with SAPS who underwent acromioplasty. It was hypothesized that adding IFT electro-massage to the usual care intervention would achieve higher effectiveness than the sole use of the usual care regime. 

## 2. Methods

### 2.1. Study Design 

A single-blinded (the evaluator assessing the outcome measures remained blinded to the participants’ allocation group) randomized controlled trial was carried out. The Consolidated Standards of Reporting Trials (CONSORT) statement and checklist were followed. The research protocol was conducted in accordance with the Declaration of Helsinki statement of ethical, legal, and regulatory principals to provide guidance for health-related research involving human subjects. The study was approved by the Ethical Research Committee of the Hospital Universitario Virgen del Rocío, Sevilla, Spain (project code CEI 2012PI/172, approval date: September 26th 2012), and prospectively registered (Clinical Trials.gov, Identifier NCT03338283). All participants provided written informed consent.

### 2.2. Participants

Adult patients with shoulder pain, who underwent acromioplasty in the 12 weeks before data collection, were referred by an orthopedic surgeon at a large public hospital in Southern Spain. Before surgery, SAPS was diagnosed following a positive response to clinical examination (Hawkins–Kennedy test, drop-arm test, external rotation lag sign, and empty can test) and radiologic diagnostic criteria to differentiate SAPS from other conditions (e.g., bone or joint abnormalities) [30]. A detailed description of the clinical tests can be found elsewhere [31]. For the diagnostic accuracy of clinical examination, a negative response to the Hawkins–Kennedy test appears to rule out SAPS (pooled sensitivity and specificity, 79% and 59%, respectively) [32]. The drop-arm test or the external rotation lag sign (specificity, 90–97%) are likely to rule in SAPS when positive [30], and the empty can test is a reliable and helpful tool to confirm subacromial impingement syndrome (87% specificity) [33]. Overall, the combination of imaging features and clinical tests can help to confirm the presence of SAPS [30]. Acromioplasty was considered a feasible intervention for patients between 20 and 80 years, with anterior shoulder pain lasting more than three months [34], and who received previous conservative treatment (manual therapy, pharmacological treatment, and use of corticosteroid injections) with no satisfactory results [35]. Those participants with a self-reported pain intensity ≥30mm in the visual analogue scale (VAS), and a score <45 points on the personal psychological apprehension scale (PPAS) [36], were invited to participate. The PPAS is a valid, reliable, and simple-to-handle tool to assess the subjects’ apprehension to receive electrical stimulation therapy [37]. The exclusion criteria were as follows: Any contraindication to the use of IFT (Table 1) [38,39]; previous cervical spine or shoulder surgery; a history of neurological or mental illnesses; diagnosed central or peripheral nervous system diseases [23]; concomitant fracture in the neck/shoulder; altered sensitivity to tactile stimuli or loss of sensation in the neck/shoulder or upper extremity [6]; concomitant radiological diagnosis of osteoarthritis of the glenohumeral or acromioclavicular joints; fibromyalgia or rheumatoid arthritis [23]; having received injections of corticoids or hyaluronic acid following surgery; symptoms of frozen shoulder [40]; impaired cognition or communication; and being involved in an on-going medico-legal dispute.

### 2.3. Study Protocol

An external website (http://www.randomization.com) was used to complete the randomization schedule for treatment order, considering a 1:1 ratio distribution of participants in the study groups (IFT electro-massage and control group). An external assistant safeguarded the randomization sequence and prepared sealed opaque envelopes concealing the treatment order allocation. Following baseline allocation, demographic and clinical data were initially collected. A blinded evaluator collected all measurements at baseline and immediately after the last treatment session. The treatment protocol consisted of a two-week intervention regime. Three treatment sessions, each lasting around 70–85 min, were made per week and supervised by a physiotherapist with more than 15 years of clinical experience.

### 2.4. Outcome Measures

Participants were asked to rate their worst shoulder pain intensity during the last 24 h using a 100-mm VAS, with 0 denoting “no pain” and 100 denoting “extreme and unbearable pain” [41]. Minimal clinically-important differences for the VAS are based on a 15–20% change [42], or a decrease above 14 mm [43], following intervention. 

The upper limb functionality was evaluated using the Constant-Murley score, which consists of a 100-point scale, with final values representing different functional levels: excellent (>80), good (65–79), medium (50–64), and bad (<50) [44]. The minimal detectable change for the Constant-Murley score has been set at 17 points for individuals with subacromial impingement syndrome [45]. 

The Simple Goniometer iPhone® app (version 1.1, Ockendon.net, Oswestry, England) was used to assess the shoulder pain-free passive range of movement. An iPhone® 3GS, iOS 4.3.5 (Apple, Cupertino, CA, USA) was fixed to the participants’ arm with an armband bracelet (Kalenji, Villeneuve d’Ascq, France). The recordings of shoulder range of motion were made twice (2-min break between assessments), using the average value of the two measures for further analysis. Before assessments, participants were asked to stop the evaluator when they started to feel low-intensity pain during movement (below 20 mm in the VAS). To evaluate shoulder flexion and extension, participants were seated with back support and no armrests, and the iPhone® was fixed on the lateral side of the arm (2 cm proximal to the glenohumeral joint). For shoulder abduction, participants kept the same position, and the iPhone® was placed on the ventral side of the arm (2 cm proximal to the glenohumeral joint). Shoulder adduction was assessed with the participant lying supine, with 90 degrees of shoulder flexion (or the maximum possible pain-free flexion), and with the iPhone® placed on the ventral side of the arm. Internal and external rotation were evaluated with participants in supine, with 90 degrees of shoulder abduction (or the maximum possible pain-free abduction), 90 degrees of elbow flexion, forearm in neutral position, and the iPhone® was placed in the ventral side of the arm (2 cm proximal to the glenohumeral joint). During all assessments, the evaluator applied gentle pressure to the arm or forearm until the edge of movement was reached [46], and maximum caution was taken to minimize the scapular motion by keeping the shoulder and back in contact with the back support. A smartphone inclinometer or virtual goniometer is an easy-to-use, valid, and reliable tool, comparable to other clinical methods, to assess shoulder range of motion in healthy subjects and in individuals with shoulder disorders [46,47,48]. The Simple Goniometer iPhone® app has shown to be reliable and possesses concurrent validity [49].

### 2.5. Interventions

**Figure 1:**
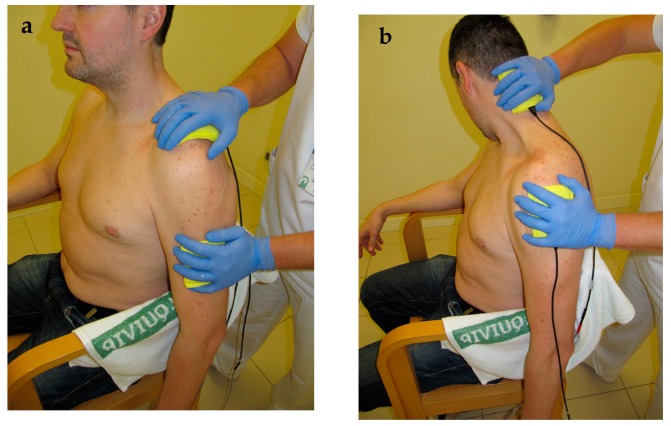
Interferential therapy electro-massage alone (**a**) or combined with stretching (**b**).

Participants in the control group underwent an usual care protocol involving: Fifteen minutes of transcutaneous infrared thermotherapy (INFRA-2000, Enraf-Nonius BV, Rotterdam, The Netherlands) [50]; 35 min of active, self-assisted, and isometric exercise therapy [51,52]; 20 min of manual therapy to retrain scapulohumeral movement and to provide soft and pain-free shoulder traction [51]; and 5 min of pulsatile ultrasound (Sonopuls 490®, Enraf-Nonius BV, Rotterdam, The Netherlands) over the acromium and scapulohumeral area, with a 5 cm^2^ head, and using a frequency of 3 Mhz and a power of 1.2 w/cm^2^. For participants in the IFT electro-massage group, a 15-min IFT electro-massage over the neck-shoulder and the glenohumeral joint was added in every treatment session to the usual care treatment previously described. A bipolar application, using a carrier frequency of 4000 Hz at constant voltage and an amplitude-modulated frequency of 100 Hz, was administered. The current intensity was set at a medium-high level, but always adapted to individual tolerance, to achieve a “strong but comfortable tingling” without evoking visible muscle twitches [28]. Two rubber electrodes (6 × 8 cm) were fitted inside sponges of equal size. The sponges were dampened with hot water to avoid unpleasant sensations and to allow a normal sliding over the skin during the electro-massage [23]. Some needles with hot water were prepared to dampen the sponges during the procedure, if required. The physiotherapist wore vinyl gloves and moved the sponges over the neck, shoulder and scapular areas. Occasionally, the therapist performed slight traction of the glenohumeral joint, and stretching of the neck-shoulder muscles (e.g., upper trapezius and levator scapulae) while administering the IFT (Figure 1).

### 2.6. Statistical Analysis 

The sample size calculation was based on detecting: (1) a 15% change in self-reported pain intensity [42]; and (2) a 17-points difference in the Constant-Murley score in the comparison between groups after intervention [45]. Taking into account a one-tailed hypothesis, an alpha value of 0.05, a desired power of 90%, and a high effect size (d = 0.8), 28 participants were required per study group (G*Power, version 3.1.9.2, Heinrich Heine University, Düsseldorf, Germany). 

Statistical processing of the data was carried out using the PASW Advanced Statistics (SPSS Inc, Chicago, IL, USA), version 24.0. Data were reported as mean (standard deviation), and confidence intervals (95% CI). The Shapiro–Wilk test was used to test the normal distribution of the study variables. Differences in the outcome measures were detected using a repeated measures analysis of variance (ANOVA), with the group (IFT electro-massage or control) as the between-subjects factor, and time (baseline or immediately after intervention) as the within-subjects factor. Post-hoc comparisons (Bonferroni) were performed for significant effects. Eta-squared (η^2^) was used to calculate the effect size (small, 0.01 ≤ η^2^ < 0.06; medium, 0.06 ≤ η^2^ < 0.14; and large, η^2^ > 0.14). Statistical significance was set at a *p* value < 0.05.

## 3. Results

**Figure 2:**
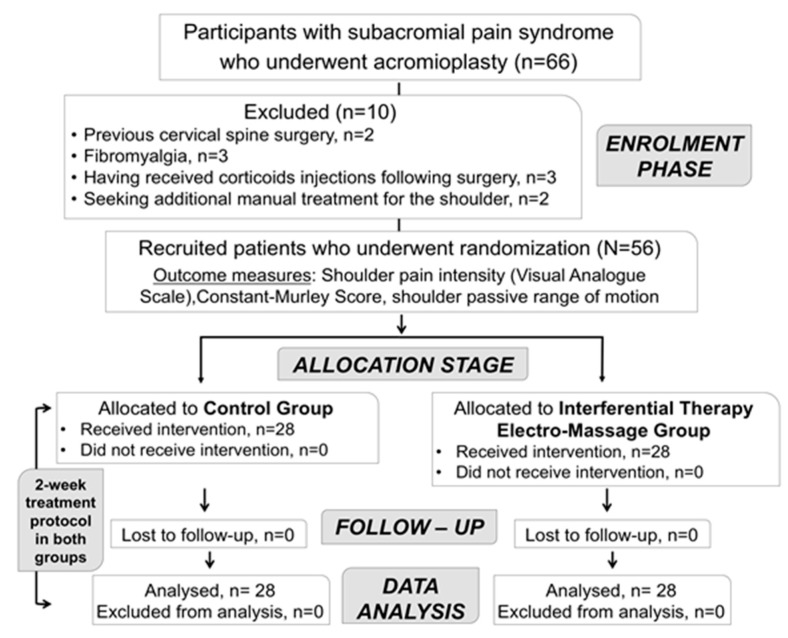
CONSORT flowchart diagram of study participants.

Sixty-six individuals who underwent acromioplasty were assessed for eligibility between December 2017 to April 2018. Finally, fifty-six participants (30 females, 53.6%), aged between 23 to 76 years (mean age ± SD, 49.6 ± 12.4), met the eligibility criteria and were recruited. There were no adverse reactions or dropouts during the study protocol (Figure 2). 

Table 2 lists the baseline characteristics of participants in the study groups. At baseline, there were no between-group differences for any study variable (all, *p* > 0.05), except for participants’ height (*p* = 0.029).

Table 3 includes the baseline, post-intervention scores, and the mean differences in the within-group and between-group comparisons for all outcome measures. Both interventions significantly improved pain perception, upper limb functionality and shoulder passive range of motion in all directions (all, *p* < 0.001). For the between-group analysis of the mean score changes after intervention, the ANOVA revealed a significant group effect, for those included in the IFT electro-massage group, for the decrease in shoulder pain intensity (F = 29.82; *p* < 0.001; η^2^ = 0.35), the improvement in the Constant-Murley score (F = 29.45; *p* < 0.001; η^2^ = 0.35), and the increase in pain-free passive shoulder flexion (F = 21.51; *p* < 0.001; η^2^ = 0.28), abduction (F = 7.77; *p* = 0.007; η^2^ = 0.12), internal rotation (F = 31.97; *p* < 0.001; η^2^ = 0.37), and external rotation (F = 8.26; *p* = 0.006; η^2^ = 0.13). There were no differences between groups for shoulder extension (F = 0.39; *p* = 0.531; η^2^ = 0.007) and adduction (F = 0.92; *p* = 0.340; η^2^ = 0.017).

## 4. Discussion

The present findings demonstrated that including IFT electro-massage in a two-week usual care protocol, combining manual therapy, exercises, thermotherapy, and ultrasound, achieved better immediate results on shoulder pain intensity, upper limb function, and pain-free passive range of movement (except for shoulder extension and adduction), compared with the sole use of the usual care regime, in adults with SAPS who underwent recent shoulder surgery. 

The decrease in shoulder pain intensity and the improvement in upper limb functionality was significantly higher, with a high effect size, for participants who received IFT electro-massage, although individuals in both groups reduced their shoulder pain after the two-week protocol above the minimum clinically important difference for the VAS [42,43]. On the contrary, changes in the Constant-Murley score surpassed the 17-point clinically relevant threshold [45] only for those in the IFT electro-massage group. The passive shoulder range of movement increased by 20–40% in the control group, and by 30–70% in the IFT electro-massage group. The differences between groups for shoulder pain-free passive mobility achieved a high effect size for shoulder flexion and internal rotation and a medium effect size for shoulder abduction and external rotation. To date, there has been a single previous study investigating the effects of IFT electro-massage [23]. This former trial used IFT as a sole intervention in individuals with chronic low-back pain and concluded greater improvements on pain, disability, and quality of life, compared to the use of superficial massage. These positive effects were explained based on the purported capacity of IFT to stimulate cutaneous sensory nerves and evoke mild vasodilation [23]. To the author’s knowledge, this is the first study assessing the effectiveness of a multimodal intervention including IFT electro-massage in adults with post-operative shoulder pain after acromioplasty. 

There is a huge debate about the clinical impact of including IFT and other electrotherapeutic modalities for the management of chronic shoulder pain. Conflicting to the current results, the addition of IFT to exercise and/or manual therapy did not demonstrate greater clinical effects on shoulder pain and disability, compared to the use of exercise and/or manual therapy alone, in individuals with non-specific soft-tissue shoulder disorders [53], or with unilateral shoulder impingement syndrome [54]. Similarly, Nazligul et al. [28] recently concluded that IFT does not provide additional effect to a multimodal approach including cryotherapy, exercise, and non-steroidal anti-inflammatory drugs for patients with SAPS. On the contrary, it has been demonstrated that the combination of IFT with shoulder exercises [55], ultrasound, thermotherapy, and stretching [56] is effective in the management of frozen shoulders. Likewise, the use of IFT alone has shown to be clinically effective to relieve pain during movement and to increase pain-free passive shoulder mobility in hemiplegic shoulder pain [57] and, when combined with exercise therapy, seems to improve pain, function, and quality of life in individuals with shoulder impingement syndrome [58]. In the latter study, the effect of combining IFT with exercise therapy was; however, similar to that of including ultrasound or transcutaneous electrical nerve stimulation instead, in the intervention protocol [58]. Indeed, IFT seems to be a potential, although modest, effective supplement to other interventions to decrease pain, compared to control or placebo treatments, in musculoskeletal pain disorders [27]. There are; however, many controversies on this issue [25,26], and the heterogeneity and methodological problems across studies make it difficult to reach conclusive statements.

This inconclusive evidence about the impact of using IFT, alone or in addition to other conservative approaches, persists when considering other chronic musculoskeletal pain conditions, such as neck or low-back pain [59]. There are some plausible explanations to account for this issue. First, the carrier frequency of the IFT current differs among studies, and this may influence the hypoalgesic response after stimulation [60]. Second, the use of electrotherapy may evoke a long sustained placebo-induced pain relief effect [61]. In this sense, most of the previous studies investigating the role of IFT on shoulder pain have not included sham IFT as a control intervention [54,55,56,58]. Third, the clinical context and the social connection between patient and therapist seem to modulate the effect of IFT [62,63], although these aspects have been scarcely controlled in the existing literature. Finally, only one previous trial has evaluated the effects of IFT, compared to sham IFT, on post-operative pain, and range of motion in patients undergoing knee surgery [64]. Even though IFT showed positive findings on increasing range of motion, and reducing pain, medication intake, and swelling [64], more definite conclusions need to be built upon more high-quality evidence [27].

Some potential study limitations should be mentioned. First, the study did not include a sham IFT electro-massage group. Second, only immediate results after the last session of the two-week intervention protocol were collected, thus it would be highly relevant to investigate the medium and long-term follow-up effects of IFT on post-operative pain in further studies. Third, the therapist in charge of the interventions was not blinded to participants allocation group. Finally, further research is warranted to investigate if different results could be expected using different current parameters.

## 5. Conclusions

Adding IFT electro-massage to a two-week supervised usual care protocol combining manual therapy, exercises, ultrasound, and infrared thermotherapy achieved better results on decreasing shoulder pain, and improving upper limb functionality and shoulder pain-free passive range of motion, compared to usual care alone, in adults with SAPS who underwent recent acromioplasty. 

## Figures and Tables

**Table 1:** List of contraindications to the use of interferential therapy.

Contraindications
• Acute inflammation
• Pregnancy
• Use of electronic devices, including cardiac pacemakers
• Active deep vein thrombosis or thrombophlebitis
• Tumoral diseases
• Use of metal implants when the subject refers unpleasant sensations
• Untreated hemorrhagic conditions or active bleeding tissues
• Recently radiated tissues
• Active tuberculosis, infected tissues, or wounds with underlying osteomyelitis • To the neck or head in individuals with previous seizures • To anterior neck, carotid sinus, over the eyes, or reproductive organs

**Table 2:** Baseline characteristics of participants in the study groups (mean ± standard deviation, or in frequency percentages).

Variable	Intereferential Therapy Electro-Massage Group (*n* = 28)	Control Group (*n* = 28)	*p* Value
Mean age (years)	47.2 ± 11.6	51.9 ± 13.1	0.159
Sex (female) % (*n*)	42.9% (12)	64.3% (18)	0.111
Height (cm)	170.18 ± 9.21	164.64 ± 9.27	0.029
Weight (kg)	80.53 ± 12.72	75.71 ± 15.44	0.208
Body mass index (kg/m^2^)	27.76 ± 3.37	27.93 ± 5.08	0.884
Arthroscopy surgery % (*n*)	85.7% (24)	89.3% (25)	0.689
Days after surgery *	42 (21–58)	51 (18–62)	0.221
Affected shoulder; right % (*n*)	50% (14)	57.1% (16)	0.595
PPAS	34.21 ± 4.74	33.54 ± 9.78	0.743
Visual analogue scale (mm)	69.82 ± 16.74	65.71 ± 20.75	0.419
Constant-Murley score (0–100)	29.68 ± 10.4	29.71 ± 12.24	0.991
Shoulder flexion (°)	103.61 ± 30.89	107.07 ± 32.53	0.684
Shoulder extension (°)	40 ± 10.79	40.18 ± 13.3	0.956
Shoulder abduction (°)	84.43 ± 27.5	84.25 ± 29.56	0.981
Shoulder abduction (°)	34.5 ± 12.08	30.86 ± 9.78	0.221
Shoulder internal rotation (°)	29.32 ± 14.75	32.21 ± 8.86	0.440
Shoulder external rotation (°)	59 ± 17.22	62.96 ± 20.74	0.378

* Median and interquartile range. PPAS—personal psychological apprehension scale.

**Table 3:** Baseline, post-intervention values, and mean score changes after intervention of the outcome measures; mean ± standard deviation (95% confidence interval).

	Baseline	After the Two-Week Intervention	Within-Group Changes after Intervention	Between-Group Mean Changes
**Visual Analogue Scale (mm)**				
IFT Electro-Massage Group	69.82 ± 16.74	32.68 ± 13.64	–37.14 ± 13.22 (–42.27 to –32.01) *	–18.92 ± 3.46 (–25.8 to –11.97) ^†^
Control Group	65.71 ± 20.75	47.5 ± 22.95	–18.21 ± 12.71 (–23.14 to –13.28) *	
**Constant-Murley Score (0–100)**				
IFT Electro-Massage Group	29.68 ± 10.41	56.07 ± 10.96	26.39 ± 5.9 (24.1 to 28.68) *	10.71 ± 1.97 (6.74 to 14.68) ^†^
Control Group	29.71 ± 12.24	45.39 ± 13.82	15.67 ± 8.61 (12.33 to 19.01) *	
**Shoulder Flexion (**°**)**				
IFT Electro-Massage Group	103.61 ± 30.89	146.86 ± 22.1	43.25 ± 16.75 (36.75 to 49.74) *	19.32 ± 4.16 (10.96 to 27.67) ^†^
Control Group	107.07 ± 32.53	131 ± 25.93	23.92 ± 14.32 (18.37 to 29.4) *	
**Shoulder Extension (**°**)**				
IFT Electro-Massage Group	40 ± 10.79	52 ± 9.86	12 ± 7.21 (9.2 to 14.79) *	1.21 ± 0.92 (–2.64 to 5.07)
Control Group	40.18 ± 13.3	50.96 ± 10.38	10.78 ± 7.21 (7.99 to 13.57) *	
**Shoulder Abduction (**°**)**				
IFT Electro-Massage Group	84.43 ± 27.5	112.18 ± 29.1	37.75 ± 15.86 (31.6 to 43.9) *	12.25 ± 4.39 (3.43 to 21.06) ^†^
Control Group	84.25 ± 29.56	109.75 ± 30.1	25.5 ± 17.01 (18.9 to 32.09) *	
**Shoulder Adduction (**°**)**				
IFT Electro-Massage Group	34.5 ± 12.08	45.54 ± 10.87	11.03 ± 7.27 (8.21 to 13.85) *	–2.25 ± 2.33 (–6.93 to 2.43)
Control Group	30.86 ± 9.78	44.14 ± 12.94	13.28 ± 10 (9.4 to 17.16) *	
**Shoulder Internal Rotation (°)**				
IFT Electro-Massage Group	29.32 ± 14.75	50.61 ± 13.31	21.28 ± 7.71 (18.29 to 24.27) *	10.5 ± 1.85 (6.77 to 14.22) ^†^
Control Group	32.21 ± 8.86	43 ± 11.2	10.78 ± 6.09 (8.42 to 13.14) *	
**Shoulder External Rotation (°)**				
IFT Electro-Massage Group	59 ± 17.22	82.5 ± 10.94	23.5 ± 13.82 (18.14 to 28.86) *	9.46 ± 3.2 (2.86 to 16.06) ^†^
Control Group	62.96 ± 20.74	77 ± 16.63	14.03 ± 10.61 (9.92 to 18.15) *	

* Indicates significant differences in the within-group comparisons (all, *p* < 0.001). ^†^ Indicates significant differences in the between-group comparisons. IFT—interferential therapy.
